# Epigenetic Repeat-Induced Gene Silencing in the Chromosomal and Extrachromosomal Contexts in Human Cells

**DOI:** 10.1371/journal.pone.0161288

**Published:** 2016-08-15

**Authors:** Sho-hei Mitsuda, Noriaki Shimizu

**Affiliations:** Graduate School of Biosphere Science, Hiroshima University, Higashi-hiroshima, Hiroshima, 739-8521, Japan; Texas A&M University, UNITED STATES

## Abstract

A plasmid bearing both a replication initiation region and a matrix attachment region is spontaneously amplified in transfected mammalian cells and generates plasmid repeats in the extrachromosomal double minutes (DMs) or the chromosomal homogeneously staining region (HSR). Generally, the repeat sequences are subject to repeat-induced gene silencing, the mechanism of which remains to be elucidated. Previous research showed that gene expression from the same plasmid repeat was higher from repeats located at DMs than at the HSR, which may reflect the extrachromosomal environment of the DMs. In the current study, plasmid repeats in both DMs and HSR were associated with repressive histone modifications (H3K9me3, H3K9me2), and the levels of repressive chromatin markers were higher in HSR than in DMs. Inactive chromatin is known to spread to neighboring regions in chromosome arm. Here, we found that such spreading also occurs in extrachromosomal DMs. Higher levels of active histone modifications (H3K9Ac, H3K4me3, and H3K79me2) were detected at plasmid repeats in DMs than in HSR. The level of DNA CpG methylation was generally low in both DMs and HSR; however, there were some hypermethylated copies within the population of repeated sequences, and the frequency of such copies was higher in DMs than in HSR. Together, these data suggest a “DNA methylation-core and chromatin-spread” model for repeat-induced gene silencing. The unique histone modifications at the extrachromosomal context are discussed with regard to the model.

## Introduction

Gene amplification of oncogenes or drug-resistance genes can play a pivotal role in mammalian carcinogenesis. The amplified genes are located either on the extrachromosomal double minutes (DMs) or on a homogeneously staining region (HSR), as reviewed in [1–3]. DMs are acentric, atelomeric, autonomously replicating chromatids composed of circular megabase-sized DNA.

Previously, we found that a plasmid with a mammalian replication initiation region (IR) and a matrix attachment region (MAR) efficiently initiated gene amplification in transfected cells, and that DMs and HSR were generated in the transfected cells [4,5]. The amplification mechanism was determined as follows. The IR/MAR plasmid was initially maintained extrachromosomally in the transfected cells, and multimerized to form a large circular molecule in which the plasmid was arranged as a direct repeat [5]. If multimerization was extensive, the large circular molecules appeared as separate cytogenetically detectable DMs. If cells contained pre-existing DMs, the plasmid repeat efficiently recombined with these DMs [6]. If the plasmid repeat integrated into the chromosome arm, a breakage-fusion-bridge cycle (BFB) was initiated, and a large HSR composed solely of plasmid repeats was formed in human COLO 320 cells [5–7] or a fine ladder-type HSR was formed in hamster CHO DG44 cells [8,9].

In general, repeat sequences are prone to silencing by the “repeat-induced gene silencing” (RIGS) mechanism [10–12]. RIGS is crucial for the maintenance of genome integrity, as it plays a role in transposon inactivation and the establishment of constitutive heterochromatin at the centromere and telomere. The RNA interference (RNAi) pathway was suggested to be responsible for the formation of heterochromatin at the centromeric satellite repeats in many organisms [13–15]. However, the RNAi pathway was not involved in heterochromatinization of transgene arrays in mammalian cells [16].

Our previous research showed that recombinant protein production in human HEK293T [17] or hamster CHO DG44 cells [18,19] was enhanced by the IR/MAR amplification technology. However, as the repeated sequence was frequently silenced, the amount of expressed RNA was less than would be expected from the amplified gene copy number [20]. To overcome this drawback, we recently isolated a highly AT-rich and CpG-poor human genomic sequence (B-3-31) that alleviated RIGS [21].

Natural DMs and HSR associated with particular cancers can carry the same sequences, as shown by an experiment in which a DM-paint probe also painted the HSR in the same cancer cell line [22]. DMs and HSR were also found to co-exist in cancer cells [23], and DMs and HSR were inter-convertible in cancer cells *in vivo* [24]. The natural amplicons observed in cancer cells were not simple repeats [25], and the gene involved was not a target of RIGS [20]. This contrasts with artificial DMs and HSR, which shared the same plasmid repeat. DMs and HSR exhibit clear differences in their replication, transcription, and repair mechanisms. For example, natural DMs and HSR in the same cell were replicated at the early and late S phase of the cell cycle, respectively [22]. Gene expression from plasmid repeats was higher with artificial DMs than with artificial HSR [20]. IR/MAR plasmids and natural DMs actively recombined with one another [5,6,26]. Furthermore, if DMs received DNA damage, multiple DMs became aggregated, resulting in segregation errors at the next mitosis [27]. These many unique features of DMs might reflect the localization of DMs in the extrachromosomal space and their inability to enter the chromosomal territory [28].

There are numerous extrachromosomal DNA elements of endogenous, exogenous, and artificial origin, and it is important to understand the extrachromosomal environment in which these elements reside. The initial question of this study was to ask whether epigenetic histone modifications and DNA methylations differed between DMs and HSR composed of the same sequence repeat. Addressing this question led to the development of a model that described how a repeat sequence might be silenced in the chromosomal and extrachromosomal contexts.

## Materials and Methods

### Cells and culture

Human colorectal carcinoma COLO 320DM (CCL-220) and COLO 320HSR (CCL-220.1) cell lines were originally derived from the American Type Culture Collection (ATCC), and cloned by limiting dilution [29]. Clones COLO 320DM (#3) and COLO 320HSR (#21) contained DMs and HSR, respectively, where many genes including c-*myc* gene were amplified. The sequence of DMs in the COLO 320DM line was described previously [25].

COLO 320 clone 12 (DM) and clone 22 (HSR) cell lines were obtained by transfecting pSFVdhfr plasmid DNA into COLO 320DM (#3) cells [5]. The plasmid contained an IR/MAR sequence from hamster *DHFR Ori ß*, and it was therefore amplified in the transfected cells. Amplified plasmids integrated into pre-existing DMs, or generated large chromosomal HSR composed solely of plasmid sequences, in clones 12 and 22, respectively. Cells were cultured in RPMI 1640 supplemented with 10% fetal calf serum.

### Fluorescence *in situ* hybridization

Plasmid pSFVdhfr was used to prepare a probe for detection of the plasmid sequence in clone 12 (DM) and clone 22 (HSR) cells [5]. Cosmid DNA (*c-myc*) was used to detect natural amplicons in COLO 320DM and COLO 320HSR cells [6]. Probes were DIG- or biotin-labeled using the BioPrime DNA Labeling System (Invitrogen) with or without 10×DIG DNA Labeling Mixture (Roche Lifescience Inc.). The probe was hybridized to metaphase chromosome spreads, and hybridized DIG- and biotin-labeled probes were detected using anti-Digoxigenin-Fluorescein Fab fragments (Roche) or Streptavidin Alexa Fluor 594 conjugate (Invitrogen), respectively.

### Chromatin immunoprecipitation

Chromatin was isolated according to a standard protocol [30] with some modification. In brief, logarithmically growing 1×10^7^ cells were fixed with 1% paraformaldehyde (PFA) for 5 min at room temperature, and fixation was then stopped by adding glycine to 350 mM. Cells were then washed in PBS(-) and treated with 10 ml of NP-40 buffer (10 mM Tri-HCl pH 8.0, 10 mM NaCl, 0.5% NP-40) for 10 min at room temperature. Cells were collected by centrifugation and suspended in 100 μl of SDS lysis buffer (50 mM Tris-HCl, pH 8.0, 1% SDS, 10 mM EDTA). After suspension, 400 μl of ChIP dilution buffer (50 mM Tris-HCl pH 8.0, 167 mM NaCl, 1.1% Triton X-100, 0.11% sodium deoxycholate (Nakarai Task, Co.), 1× proteinase inhibitor cocktail (Roche)) was added. Chromatin was sonically sheared to a median size of 500 bp using an EpiShear Sonicator (Active Motif). A 50 μl aliquot was retained for isolation of control “input” DNA.

For chromatin immunoprecipitation, 50 μl of Dynabead Protein G Magnetic beads (Dynal) was washed and resuspended in PBS(-)/0.02% Tween 20 using a magnetic stand (Millipore). The following antibodies (3–10 μg per sample) were bound to the beads: mouse monoclonal anti-histone H3K9me3 antibody (Active Motif, 61013), mouse monoclonal anti-histone H3K9ac antibody (Active Motif, 61251), mouse monoclonal anti-histone H3K9me1 antibody (Active Motif, 39681), mouse monoclonal anti-histone H3K9me2 antibody (Active Motif, 39683), rabbit polyclonal anti-histone H3K4me3 antibody (Active Motif, 39159), rabbit polyclonal anti-histone H3 (dimethyl K79) antibody (Abcam, ab3594), and mouse (G3A1) mAb IgG1 Isotype Control (Cell Signaling, 5415S). The sheared chromatin was mixed with the antibody-protein G complex and incubated for 10 min at room temperature. The beads were washed first with ice cold 150 mM RIPA buffer (50 mM Tris-HCl pH 8.0, 150 mM NaCl, 1 mM EDTA, 0.1% SDS, 1% Triton X-100, 0.1% sodium deoxycholate), then with 500 mM RIPA buffer (as above, except with 500 mM NaCl), then with LiCl wash buffer (10 mM Tris-HCl pH 8.0, 250 mM LiCl, 1 mM EDTA, 1% sodium deoxycholate, 1% NP-40), and finally with TE (10 mM Tris-HCl pH 7.0, 1 mM EDTA). The washed beads were then suspended in ChIP elution buffer (10 mM Tris-HCl pH 8.0, 300 mM NaCl, 5 mM EDTA, 0.5% SDS). The 50 μl input sample retained after shearing was combined with 150 μl of ChIP elution buffer and SDS to a final concentration of 0.5%. Samples were de-cross-linked by incubating at 65°C overnight. De-cross-linked samples were treated with DNase-free RNase (Roche) and proteinase K (Wako co.), prior to phenol extraction/ethanol precipitation, and final resuspension of the DNA in TE.

### Real-time PCR analysis

Primers (18–24 nt, 40–60% GC, and Tm = 57–62°C) that amplified 70–170 bp regions of *GAPDH*, c-*myc*, and various sequences within the pSFVdhfr plasmid were designed using Primer 3 (http://bioinfo.ut.ee/primer3-0.4.0/) and Amplify4 (http://engels.genetics.wisc.edu/amplify/) software. Primer sequences are given in S1 Table. Template DNA was diluted by TE to a concentration of 1×10^3^ cell-equivalents/μl. PCR was performed in triplicate using THUNDERBIRD SYBR qPCR Mix (Toyobo, Co.) and the StepOnePlus Real Time PCR System (Applied Biosystems). ChIP analysis results were expressed relative to input, which was calculated from the difference in Ct values between test and input DNA, and normalized to the amplification efficiency obtained from the standard curve.

### Bisulfite sequencing

Genomic DNA was purified according to a standard protocol. Bisulfite reactions were performed using an EpiTect Fast DNA Bisulfite kit (QIAGEN) according to the manufacturer’s recommended protocol. Bisulfite-treated DNA was purified using a column supplied with the kit. CpG island prediction and PCR primer design were performed using Methprimer software (http://www.urogene.org/cgi-bin/methprimer/methprimer.cgi). The bisulfite reaction converted unmethylated C to U. Correspondingly, PCR primers were designed according to the target sequence, with C (except for CpG, assuming it was methylated) replaced by T. Each primer contained no more than three CpG. Primers were 25–40 nt in length, and the resulting amplicons were <300 bp. PCR reactions were performed using bisulfite-treated DNA, forward and reverse primers designed as above, and Blend Taq-plus DNA polymerase (Toyobo, Co.). PCR products were ligated into the pGEM-T Easy Vector (Promega), and transformed into *E*. *coli* DH5α host cells. Cloned inserts were PCR amplified directly from *E*. *coli* colonies using primers (pGEM-T(+) and pGEM-T(-)) that flanked the vector insertion site. PCR products were purified, then sequenced using the pGEM-T(+) or pGEM-T(-) primer with a BigDye Terminator v3.1 Cycle Sequencing kit and an ABI PRISM 310 Genetic Analyzer (Applied Biosystems). The methylation status of the sequences was analyzed using QUMA (QUantification tool for Methylation Analysis; http://quma.cdb.riken.jp/top/quma_main_j.html) software.

## Results

### Repressive histone modifications accumulate at higher levels in HSR plasmid repeats than in DM plasmid repeats

**Fig 1:**
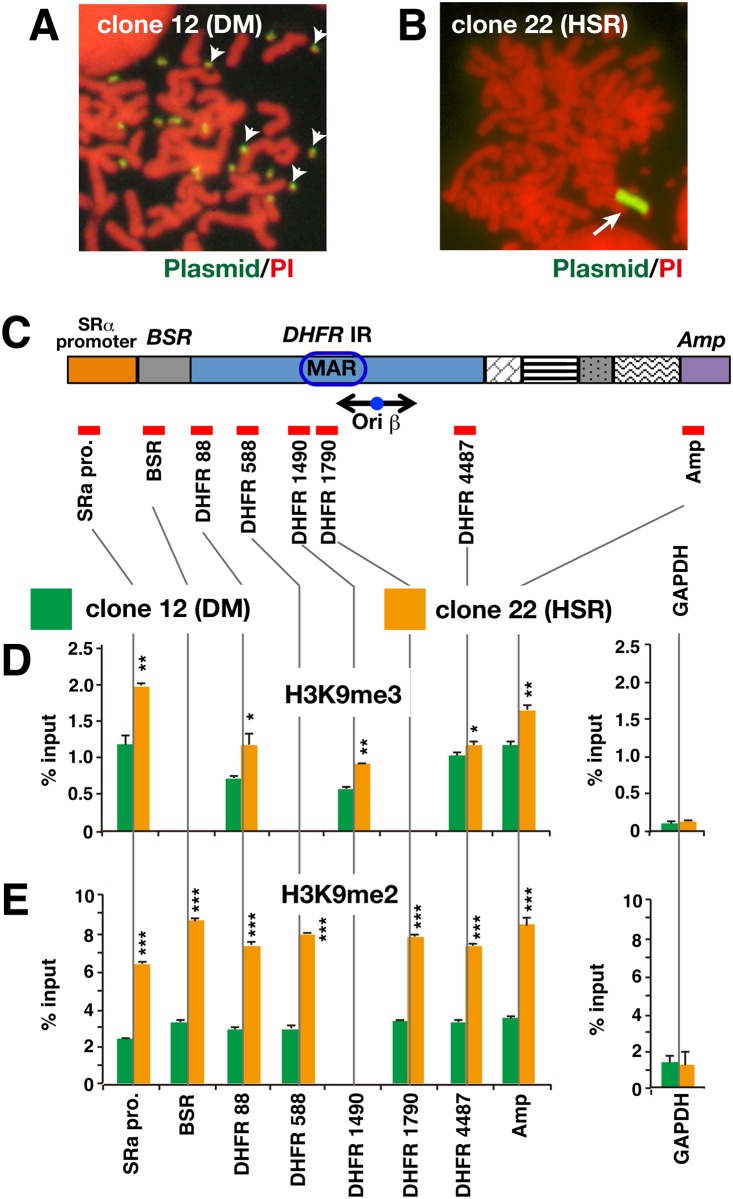
Repressive histone modifications at plasmid repeats in DMs and HSR. (A, B) Metaphase chromosome spreads from COLO 320 clone 12 (DM) and clone 22 (HSR) were hybridized with a probe prepared from plasmid pSFVdhfr. The hybridized probe was detected as green fluorescence, and DNA counterstained by propidium iodide (PI) is shown in red. The hybridized plasmid probe appears at the multiple DMs (A; arrowheads) and at the chromosomal HSR (B; arrow). (C) The structure of the pSFVdhfr plasmid is depicted, with the positions of regions used for real-time PCR analysis shown as red bars. (D, E) Chromatin from clone 12 (DM; green bars) and clone 22 (HSR; orange bars) was precipitated using antibody specific to H3K9me2 or H3K9me3. Sequences along the plasmid were quantitated in immunoprecipitated DNA using real-time PCR. The housekeeping gene *GAPDH* was used as a control. Results (mean +/- SD; n = 3) are shown as a percentage of input chromatin. The statistical significance of the difference between clone 12 (DM) and clone 22 (HSR) is analyzed by student’s t-test. ***; p<0.001, **; p<0.01, *; p<0.1.

Previously, we isolated human COLO 320 clone 12 (DM) and clone 22 (HSR) by transfecting the IR/MAR-bearing pSFVdhfr plasmid into human COLO 320DM (#3) cells [5]. Fig 1A and 1B show representative metaphase images for clone 12 and clone 22 cells, respectively. The probe prepared from plasmid pSFVdhfr was found to hybridize to multiple DMs in clone 12 (DM) and to a single large HSR in clone 22 (HSR). Our previous study showed that, in clone 12, low double-digit numbers of tandem repeat plasmid copies integrated to pre-existing DMs in COLO 320DM cells. By contrast, the large chromosomal HSR in clone 22 consisted of ~1000 plasmid repeats [5].

To examine histone modification in DMs and HSR, chromatin was isolated from logarithmically growing cell populations and then immunoprecipitated using antibodies specific to histone H3 di- or trimethylated at K9 (H3K9me2 or H3K9me3). DNA was recovered from the precipitated chromatin and subjected to real-time PCR to quantitate the amount of sequence corresponding to positions along the pSFVdhfr plasmid (Fig 1C). A sequence from *GAPDH*, an actively transcribed housekeeping gene, was used as a control. H3K9me2 and H3K9me3 are markers of repressed chromatin and, as expected, levels of these modifications were low at *GAPDH* (Fig 1D and 1E). In addition, H3K9me2 and H3K9me3 levels were very similar between clone 12 and clone 22, reflecting *GAPDH* was unrelated to plasmid amplification. By contrast, higher H3K9me2 and H3K9me3 levels were detected at the plasmid repeat in both DMs and HSR (Fig 1D and 1E). The accumulation of repressed chromatin markers at DMs suggested that RIGS could occur in an extrachromosomal context. However, both types of histone modification were more pronounced in HSR repeats (clone 22) than in DM repeats (clone 12). When the different plasmid regions were considered, H3K9me3 was associated most extensively with the SRα promoter region, whereas H3K9me2 was evenly distributed across the whole plasmid sequence.

### Active histone modifications at plasmid repeats are found at higher levels in DMs than in HSR

Next, we examined chromatin from COLO 320 clone 12 (DMs) and clone 22 (HSR) using antibodies specific to active chromatin modifications. While trimethylation or dimethylation at H3K9 is associated with repressive chromatin, monomethylation of the same position (H3K9me1) is linked to active chromatin [31]. Trimethylation of H3 at K4 (H3K4me3) is related to transcriptional activation, and may be involved in nucleosome removal at the transcription start site (TSS) [31]. Acetylation of H3 at K9 (H3K9ac) is another well-characterized active chromatin marker.

**Fig 2:**
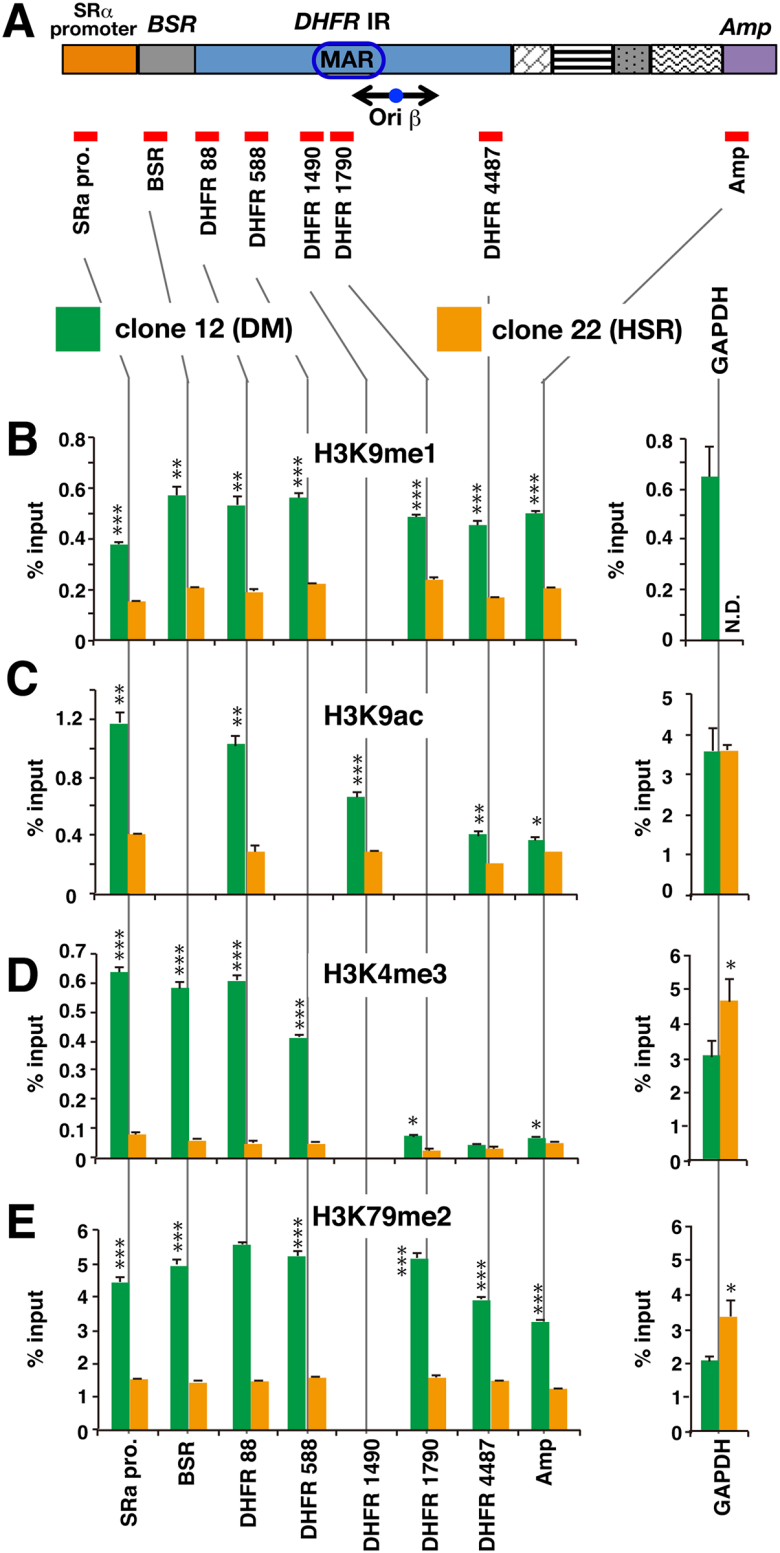
Active histone modifications at plasmid repeats in DMs and HSR. (A) The structure of the pSFVdhfr plasmid is depicted, with the positions of the amplified regions used for quantitative PCR indicated in red. Chromatin from COLO 320 clone 12 (DM) and clone 22 (HSR) was immunoprecipitated using antibodies specific to H3K9me1 (B), H3K9ac (C), H3K4me3 (D), or H3K79me2 (E). Sequences along the plasmid were quantitated in immunoprecipitated DNA using real-time PCR. The housekeeping gene *GAPDH* was used as a control. Results (mean +/- SD; n = 3) are shown as a percentage of input chromatin. The statistical significance of the difference between clone 12 (DM) and clone 22 (HSR) is analyzed by student’s t-test. ***; p<0.001, **; p<0.01, *; p<0.1.***; p<0.001, **; p<0.01, *; p<0.1.

The results of the active chromatin analysis are shown in Fig 2. H3K9me1 was detected at only a low level in both *GAPDH* and the repeated plasmid sequences in DMs or HSR. However, throughout the plasmid sequence, the H3K9me1 level was higher in DMs than in HSR (Fig 2B). Higher levels of H3K9ac and H3K4me3 were detected at the *GAPDH* gene in both clone 12 and clone 22, which was consistent with the role of *GAPDH* as a housekeeping gene (Fig 2C and 2D). Levels of both H3K9ac and H3K4me3 were very low throughout the entire plasmid region in clone 22 (HSR) cells, contrasting with much higher levels in clone 12 (DM) cells, particularly around the *BSR* locus. These results demonstrate clear differences in the levels of active chromatin modifications between DMs and HSR.

H3 dimethylated at K79 (H3K79me2) is a cell cycle-dependent modification at active genes that is commonly detected at mammalian replication initiation sites [32]. Levels of H3K79me2 modifications were much higher at DMs (clone 12) than at HSR (clone 22; Fig 2E). The highest modification level was found at the *DHFR* replication IR. These results were consistent with those above, and again indicated that active chromatin markers were found more frequently at DMs than at HSR. Furthermore, the data suggest that the IR sequence was involved in replication initiation of the amplified array in DMs.

Taken together, the results indicated that HSR chromatin was relatively more repressed (higher levels of H3K9me3 and H3K9me2) and DM chromatin was more active (higher levels of H3K9me1, H3K9ac, H3K4me3, and H3K79me2).

### C-*myc* genes amplified during carcinogenesis are associated with active chromatin irrespective of their location at DMs or HSR

**Fig 3:**
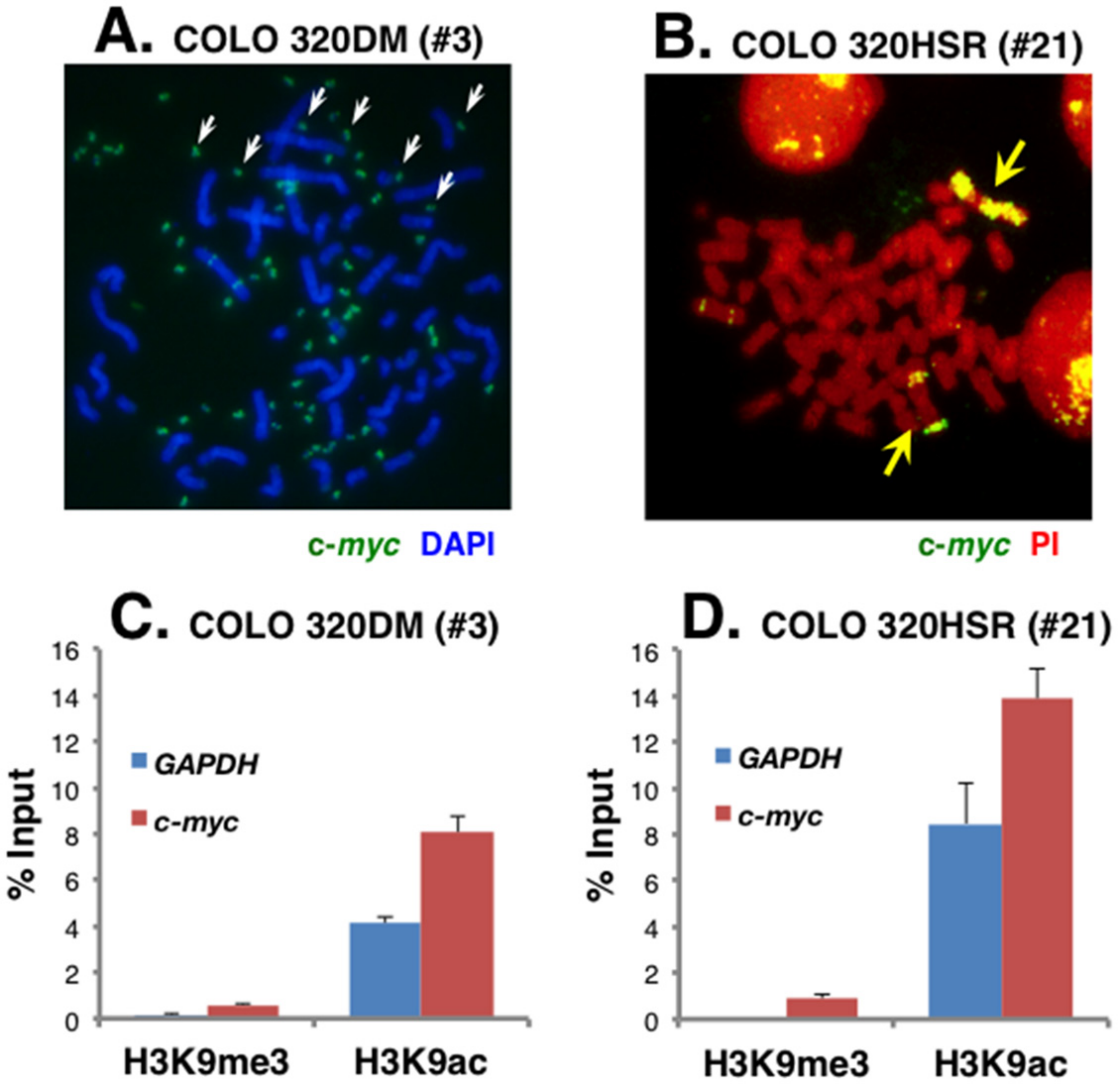
Histone modifications in DMs or HSR at c-*myc* amplified genes generated during carcinogenesis. (A, B) Metaphase chromosome spreads from COLO 320DM (#3) and COLO 320 HSR (#21) cells were hybridized to DIG-labeled c-*myc* cosmid probe. DNA was counterstained with DAPI (blue in A) or PI (red in B). The hybridized probe (green) was detected only at multiple DMs (white arrows in A) or HSRs (yellow arrows in B). Chromatin from these cells was precipitated by antibodies specific to H3K9me3 or H3K9ac. The amount of *GAPDH* or c-*myc* in the precipitated DNA was analyzed by quantitative PCR. Results (mean +/- SD; n = 3) are shown as a percentage of input chromatin.

Next, chromatin modifications were examined in COLO 320DM (#3) and COLO 320HSR (#21) cells, in which c-*myc* was amplified during carcinogenesis. Fig 3A and 3B show representative metaphase images of these cells labeled with a c-*myc* cosmid probe. Amplified c-*myc* genes localized to DMs and HSR in #3 and #21 cells, respectively. Previous research showed that the DM-paint probe prepared from COLO 320DM (#3) homogeneously painted the HSR in COLO 320HSR (#21), indicating that DMs in COLO 320DM (#3) and the HSR in COLO 320HSR (#21) shared the same amplified region [22].

Chromatin was isolated from these cells and immunoprecipitated using antibodies specific to H3K9me3 or H3K9ac. Precipitated DNA was analyzed by PCR. H3K9ac levels were high and H3K9me3 levels were low in both *GAPDH* and c-*myc* (Fig 3C and 3D). This suggested that both the housekeeping gene *GAPDH* and the amplified c-*myc* genes were embedded in active chromatin regions in both COLO 320DM (#3) and COLO 320HSR (#21) cells, indicating that DMs and HSR generated during carcinogenesis were active. This was consistent with our previous data showing that c-*myc* transcript levels were almost proportional to the c-*myc* gene number in COLO 320DM (#3) and COLO 320HSR (#21) cells [20]. The natural amplicon structures in these cells were not simple repeats, but were highly complex [25], and such complex structures may not be targeted by RIGS.

### Inactive chromatin spreads to the surrounding chromatin in the extrachromosomal context

**Fig 4:**
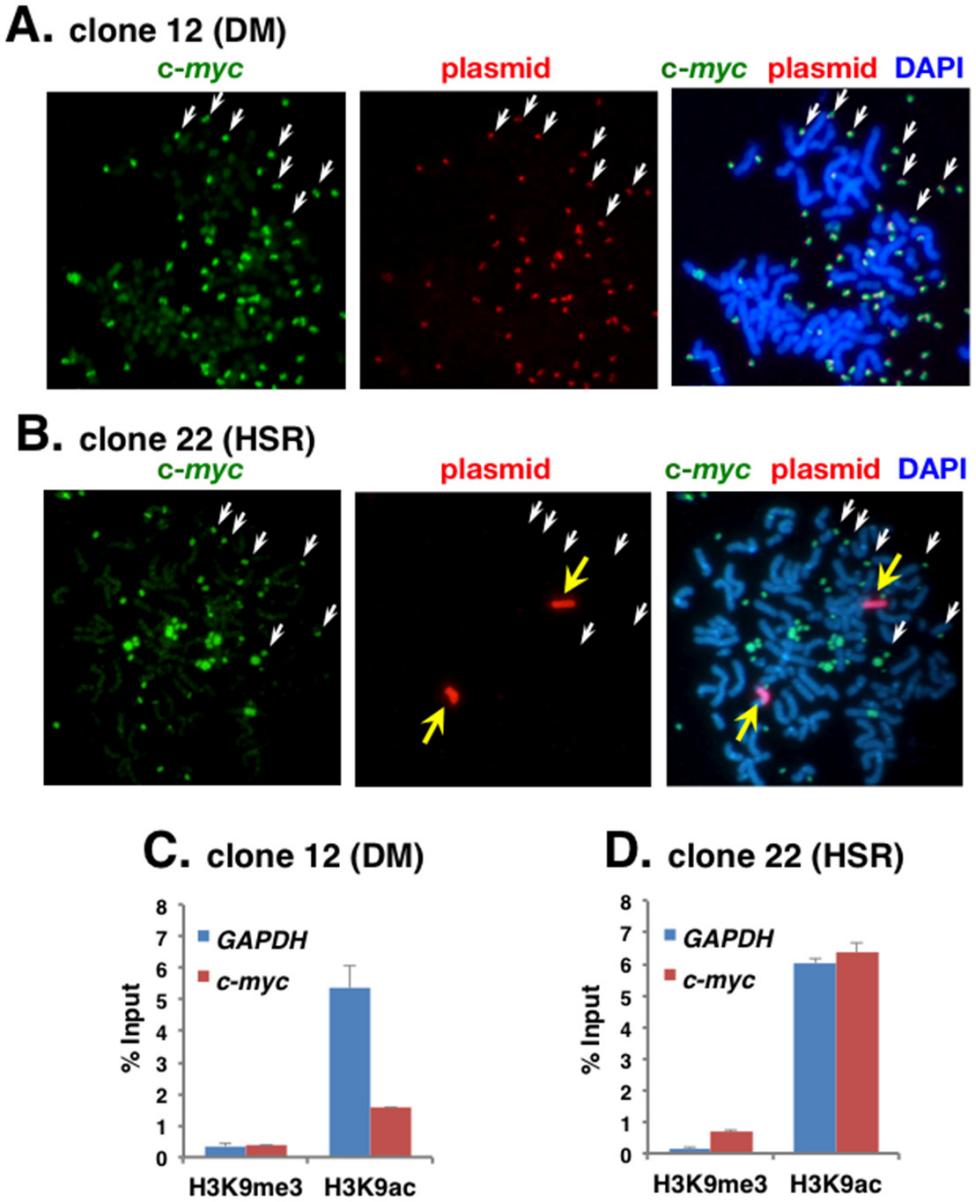
Histone modification at amplified c-*myc* sites adjacent to plasmid repeats. Metaphase chromosomes from clone 12 (DM; A) or clone 22 (HSR; B) were simultaneously hybridized with c-*myc* probe and plasmid probe, detected in green and red fluorescence, respectively. DNA was counterstained in blue (DAPI). In clone 12 (DM), the amplified IR/MAR plasmid integrated to pre-existing DMs where amplified c-*myc* was already present. In clone 22 (HSR), the amplified IR/MAR plasmid generated chromosomal HSR independently from pre-existing DMs. Chromatin was isolated from the cells and immunoprecipitated with antibodies against H3K4me3 or H3K4ac. The amount of *GAPDH* and c-*myc* in the precipitated DNA was analyzed by quantitative PCR. Results (mean +/- SD; n = 3) are shown as a percentage of input chromatin.

Plasmid pSFVdhfr and c-*myc* sequences were detected simultaneously among metaphase chromosome spreads from COLO 320 clone 12 (DM) and clone 22 (HSR) cells (Fig 4A and 4B). C-*myc* was amplified at multiple DMs in both clones, whereas plasmid sequences were amplified at DMs in clone 12 (DM) and at HSR in clone 22 (HSR). Therefore, c-*myc* and amplified plasmid colocalized at DMs in clone 12 (DM), but the two amplicons were separately located in clone 22 (HSR).

Chromatin from COLO 320 clone 12 (DM) and clone 22 (HSR) cells was immunoprecipitated using antibodies specific to H3K9me3 or H3K9ac, and *GAPDH* and c-*myc* levels were quantitated (Fig 4C and 4D). As anticipated, *GAPDH* was associated with high H3K9ac levels and low H3K9me3 levels in both clone 12 and 22. C-*myc* chromatin was also highly acetylated and rarely methylated at H3K9 in clone 22 (HSR) cells, consistent with the results shown in Fig 3. However, c-*myc* chromatin received significantly less acetylation at H3K9 in clone 12 (DM) compared to clone 22 (HSR). This suggested that c-*myc* chromatin became repressed after the neighboring insertion of the repeated plasmid array, implying that the repressed chromatin spread to the adjacent region. The spread of heterochromatin is well characterized in chromosomes, and our findings suggest that similar spreading can also occur in the extrachromosomal context.

### DM and HSR repeats are generally CpG hypomethylated, but some hypermethylated copies are present

**Fig 5:**
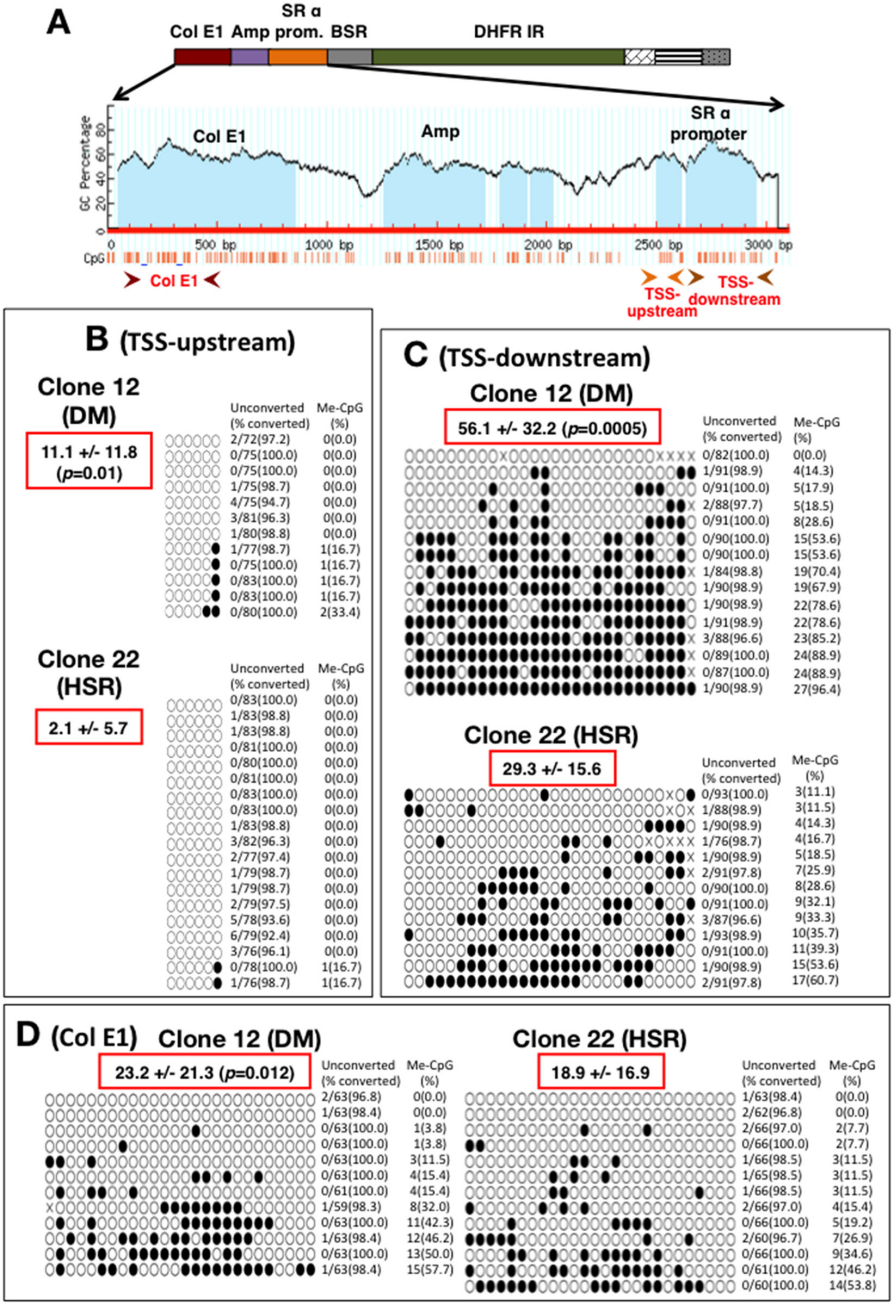
Methylation of CpG in plasmid repeats in DMs and HSR. (A) The structure of pSFVdhfr plasmid and a GC percentage plot from Col EI to the SRα promoter region are shown. The positions of CpG dinucleotides (red bars) and PCR primer pairs (red arrowheads) are shown beneath the plot. (B-D) Summary of bisulfite sequencing results. Six CpGs at TSS-upstream (B), 28 CpGs at TSS-downstream (C), and 26 CpGs at Col E1 (D) were examined in DNA from clone 12 (DM) and clone 22 (HSR) cells. White and black ovals represent unmethylated and methylated CpGs, respectively. X in panel C represents CpG, whose methylation status could not be determined. Each line represents a sequence read. The number of unconverted C (except at CpG) and the conversion percentage are given to the right of each read. The number of methylated CpG and methylation as a percentage of total CpG are noted at the far right. Mean +/- SD of the percent of methylated CpG is noted in red box. The average was usually higher for DMs than HSR, and the p value is noted in the same red box.

Next, we examined DNA CpG methylation in repeated plasmid sequences in DMs and HSR. Genomic DNA was isolated from COLO 320 clone 12 (DM) and clone 22 (HSR) and treated with bisulfite to convert unmethylated cytosine to uracil. Three CpG-rich regions inside the plasmid sequence were PCR amplified (Fig 5A): two CpG islands upstream or downstream of the TSS inside the SRα promoter, and a CpG-rich sequence at the *E*. *coli* origin of replication (Col E1). PCR products were cloned and amplified in *E*. *coli* prior to sequencing. Sequences from bisulfite-treated DNA were compared with the original sequence to identify methylated and unmethylated CpG sites.

The conversion rate of cytosine to uracil at sites other than CpG was very high (Fig 5B to 5D), indicating that bisulfite treatment was successful. CpG methylation was generally low, particularly at the short TSS-upstream region (Fig 5B). This was consistent with previous observations of low CpG methylation close to the TSS [16]. Methylation at the long TSS-downstream region was relatively high (Fig 5C), and, furthermore, methylation was higher in clone 12 (DM) than in clone 22 (HSR). This hypomethylated characteristic was contradictory to the association of repeats with repressed chromatin and the heterochromatinized state of the entire HSR [33]. However, the methylation level varied considerably between individual sequences, and some hypermethylated sequences were observed. Twenty-eight CpGs were examined at the TSS-downstream region, and the percentage range of methylated CpG was 0–96.4% for DM and 11.1–60.7% for HSR. Methylation levels at the 26 CpG examined at ColE1 were 0–57.7% for DM and 0–53.8% for HSR. Because the plasmid sequence was highly amplified in clone 12 and clone 22, heterogeneity between sequence reads probably reflected heterogeneity between the copy repeats in DMs or HSR.

## Discussion

Identical plasmid repeat sequences were associated with different chromatin modifications depending on whether the repeats were in a chromosomal or an extrachromosomal context. Specifically, compared to HSR, repeat sequences in DMs were less associated with repressive chromatin (Fig 1) and more associated with active chromatin (Fig 2). This was consistent with previous results showing that the plasmid sequence was more actively transcribed in DMs than in HSR [20]. Spreading of repressive chromatin to surrounding chromosomal regions, in the absence of a boundary element, is a well-known phenomenon [34]. Here, we showed that this phenomenon also occurred in an extrachromosomal context (Figs 3 and 4). The majority of repeat sequences at both DMs and HSR were CpG hypomethylated (Fig 5). This was consistent with hypomethylation of CpG at centromeric repeat sequences [35] or transgene arrays [16]. In addition, repeat sequences were generally hypomethylated in cancer cells (reviewed in [36]). Our data suggested that the methylation level was heterogeneous between the repeated copies. A few hypermethylated copies were observed, and the frequency of hypermethylation of the CpG island in the TSS-downstream region was higher in DMs than in HSR.

**Fig 6:**
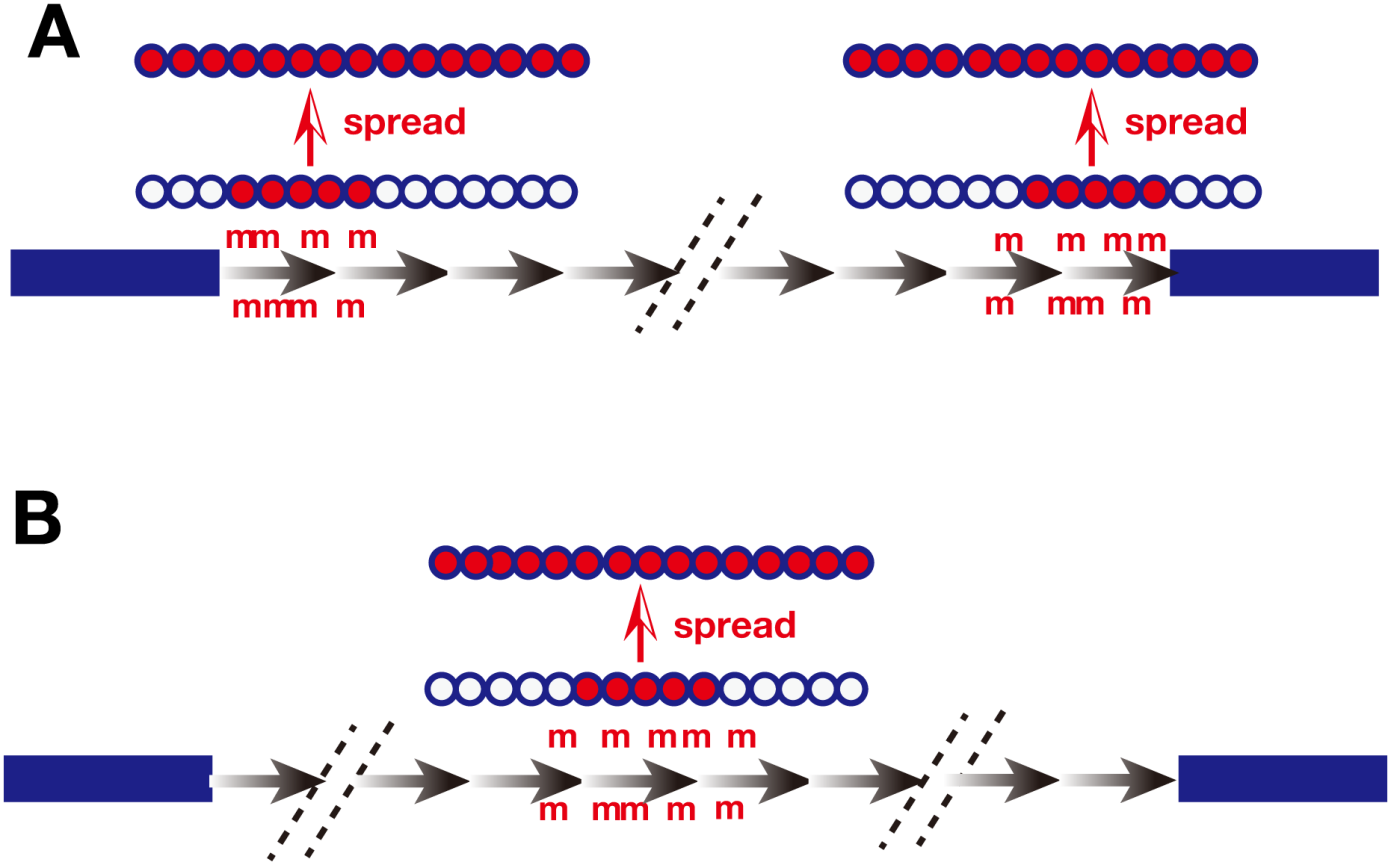
A “DNA methylation-core and chromatin-spread” model of RIGS. Repeated copies of plasmid sequences are shown with gray arrows. Restricted copies adjacent to the genomic sequence (bold navy line) at either end of the full repeat sequence (A) or internal repeat copies (B) are methylated (m) at CpG dinucleotides. Histone (circles) is then modified to the repressed state (red) at the DNA methylation site, and chromatin repression spreads in both directions along the repeats.

From our results, we hypothesized the model depicted in Fig 6. Our data suggested that CpG methylation preferentially occurred at a few restricted copies of the repeat sequences in DMs or HSR. Methylation may occur either at the end of a repeat that flanks the chromosome arm (Fig 6A) or at an internal site within the repeat (Fig 6B). The length of the repeated sequences in individual DMs was shorter than in HSR [5], and multiple DMs were found per cell, which might explain the higher frequency of hypermethylated sequences in clone 12 (DM) than in clone 22 (HSR) seen in Fig 5. Although a cause-and-effect relationship was not established, previous reports noted a tight relationship between DNA methylation and H3K9me3/H3K9me2-positive heterochromatin [37,38]. We hypothesize that heterochromatin might initially form at the limited number of CpG hypermethylated copies and then spread in both directions along the chromatin. This would establish the complete heterochromatinization of HSR [33]. Such a “DNA methylation-core and chromatin-spread” model of RIGS is consistent with our previous results using the same the IR/MAR plasmid repeat. First, gene expression from the plasmid repeat in HSR was alleviated by either sodium butyrate or 5-azacytidine [20,21], which up-regulated histone acetylation and down-regulated DNA methylation, respectively. Second, a long array of extremely AT-rich/GC-poor sequence (B-3-31) upstream of the promoter alleviated repeat-induced gene silencing [21]. Third, transcription inside the large heterochromatinized HSR was observed at only a few restricted foci, and the number of such foci decreased/increased according both to the cell cycle position and the nucleolar association of the HSR [33]. This suggested that heterochromatinization of the HSR was flexible, which could be explained by the above model. Dynamic shifts in chromatin structure might occur in response to DNA methylation landmarks acting as heterochromatin nucleation sites, and chromatin spreading might vary depending on cellular conditions.

Our results suggested that spreading of repressive chromatin might occur in the extrachromosomal context (Figs 3 and 4). However, the spreading appeared to be incomplete because, compared to HSR, DMs were associated with less repressive and more active chromatin. This was consistent with the differences between DMs and HSR seen in gene expression and replication timing. DMs reside in the inter-chromosomal space and do not enter the chromosome territory [28]. This environment may be different from that within the chromosome territory, where most of the chromosome arms reside, and may explain the incomplete heterochromatinization seen at DMs. Alternatively, we previously observed that most of the DMs were located at the nuclear periphery during the G1 phase of cell cycle, and then moved to the nuclear interior when DMs themselves were replicated at early S phase [22,39]. The nuclear periphery is usually rich in heterochromatin, with most of the euchromatin located at the nuclear interior. It is therefore likely that the nuclear localization of DMs might affect the establishment and spreading of heterochromatin in DMs.

When taken together, the data support our proposed “DNA methylation-core and chromatin-spread” model for the silencing of repeated genes. This model may be applicable to transgenes in general, because generation of transgene repeats and their silencing is frequently observed. Furthermore, we showed that histone modifications in the extrachromosomal context were unique, and might be explained by the incomplete spreading of repressive chromatin. We speculate that this unique context might be relevant to a wide variety of extrachromosomal elements of endogenous, exogenous, and artificial origin.

## Supporting Information

S1 Table Primers used in this study. Sequences of PCR primers are listed in this table.
(DOC)
