# Supplementary material for: Epigenetic Repeat-Induced Gene Silencing in the Chromosomal and Extrachromosomal Contexts in Human Cells
Source: PLoS One. 2016 Aug 15;11(8):e0161288. doi: 10.1371/journal.pone.0161288 (PMC4985131; doi:10.1371/journal.pone.0161288)
Supplement: S1 Table — Sequences of PCR primers are listed in this table. (DOC) [file pone.0161288.s001.doc]

|  | Forward | Reverse |
| --- | --- | --- |
| *GAPDH* | TACTAGCGGTTTTACGGGCG | TCGAACAGGAGGAGCAGAGAGCGA |
| SR α promoter | CTCGCATCTCTCCTTCACG | CGGTCTCGACCTGAGCTTTA |
| *BSR* | AGCGACAGAGAAGATTACAATGC | CGAAACTGCACTACCAATCGC |
| *DHFR* 88 | CTTAAATGTGACTCCCCCAGAC | CATCAGACTGGTCCCATATCC |
| *DHFR* 588 | CTGTTGCAGTGACCCAGAAATG | TTGTCACTCACTACCTGATGCC |
| *DHFR* 1490 | TTGGCATCTGAATATTCTTTTTG | TGCCTCAGTGTGATGTTTTTG |
| *DHFR* 1790 | GTTATGTTTGGTGTGGTGGTTC | CACATAGTTCAGGTTGGCCTTG |
| *DHFR* 4487 | GCATGGCTTATCTGCATCCT | ATCCATTGTGTGGCTCCTTC |
| *Amp* | GAATAAGGGCGACACGGAAATG | TCCGCTCATGAGACAATAACCC |
| c-*myc* exon 1 | ACTTACAACACCCGAGCAAG | TCTAAGCAGCTGCAAGGAGAG |

**S1 Table. Primers used in this study**
